# Challenges faced by blind patients using chronic medication at a tertiary hospital in South Africa

**DOI:** 10.4102/hsag.v27i0.1900

**Published:** 2022-12-05

**Authors:** Madan S. Poka, Jonathan T. Chanyandura, Bwalya A. Witika

**Affiliations:** 1 Department of Pharmaceutical Sciences, Faculty of Health Sciences, Sefako Makgatho University, Pretoria, South Africa; 2 Department of Pharmacy, Faculty of Health Sciences, University of Limpopo, Polokwane, South Africa

**Keywords:** blind, partially blind, visually impaired, chronic, medicine, self-administration

## Abstract

**Background:**

Difficulties faced by blind patients in using medicines are largely unknown and underexplored. This limits the ability of health providers and health policy makers to plan and provide for medicine related needs of this special group.

**Objectives:**

To describe the challenges faced by blind patients around Mankweng Hospital when taking chronic medications and to identify methods used to overcome the challenges.

**Methods:**

Quantitative cross-sectional descriptive study, where questionnaires were administered to 82 blind patients, 18 years and older, and who were on chronic medications. Data was analysed using the Statistical Package for the Social Sciences (SPSS) software.

**Results:**

Majority of participants were elderly (59%) and had partial blindness (78%). Challenges faced by participants included inability to locate and identify medication (60%), missing doses (64%), inaccurate dosing and spilling medicines (33%). A staggering 68.3% of the participants did not have specific methods to overcome challenges.

**Conclusions:**

Challenges faced by the blind and visually impaired are similar across the world. However, participants are unaware of other simple, feasible methods available in the market. Current methods used by the participants to overcome the challenges encountered are minimal or caregiver dependent. Programs may be set up at clinics, hospitals and health care centers to teach the visually impaired simple and inexpensive methods to help administer medications.

**Contribution:**

Results obtained may be used to raise awareness in health care policy makers of the under-explored challenges faced by the partially blind or completely blind patients in the use of medicines.

## Introduction

A person is regarded as blind when they are unable to see because of injury, disease or congenital condition, or lacking a sense of sight (WHO 2002). Total blindness is the inability to tell light from dark, or the total inability to see. Visual impairment, or low vision is a severe reduction in vision, that cannot be corrected with standard glasses or contact lenses, and reduces a person’s ability to perform certain or all tasks (Freeman et al. 2007). There are 285 million people with visual impairment worldwide, of which 39 million are blind and 246 million (m) have low vision (Weeraratne, Opatha & Rosa 2012). Just under 90% of these people live in developing countries such as Sri Lanka, South Africa and Brazil. In South Africa, 1.3% of the population, which is approximately about 600 000 people, are visually impaired, making visual impairment the largest disability (Sacharowitz 2005). Data from the National Guideline: Prevention of Blindness in South Africa, (Department of Health, Republic of South Africa 2002), December 2002, reported a 0.75% prevalence of blindness in the South African population. In addition, 80% of people who are blind were noted as living in rural areas. Worldwide, people of age 50 years and older, represent 65% and 82% of those with visual impairment and blindness, respectively (Mariottic & Pascolini 2012). The leading causes of blindness worldwide include cataracts, glaucoma, and age-related macular degeneration (Sacharowitz 2005). About 62% of people over age 65 years, have more than one chronic condition, and the prevalence of multiple chronic conditions increases with the increasing ageing of populations (Jaul & Barron 2017). Patients with complete or partial blindness, like other patients, should use medication as prescribed or directed in order to receive the desired therapeutic benefits. However, visual impairment comes with unique difficulties when using medication. Such difficulties include failure to identify expiry dates of the medicine, failure to recognise medication to be taken, dose inaccuracy and frequency, problems in reading labels and warnings (Kentab et al. 2015). According to Weeraratne et al. (2012), people with partial sight and people who are completely blind are potentially more likely to have unsafe practices related to medicine use. Most of the challenges that patients with blindness face, in terms of medicine use, may lead to unsafe medical practices, such as overdosing, leading to medicine toxicity, or subdosing, leading to poor therapeutic effect. These consequences may leave patients discouraged to continue taking medications, and they may decide to stop the treatment immediately when they start feeling better, which may further lead to patient noncompliance. Several strategies and methods are in use to overcome the challenges experienced by patients with visual impairment. These methods include audio prescriptions, and braille labels, where information about the medication is transcribed into audio or braille (Almukainzi et al. 2020). Such methods are expensive, and only developed countries can afford them. The rest of the world may rely on methods such as tracking the medications by placing them in alphabetical order and storing them where they are administered. Furthermore, other inexpensive methods include use of rubber bands, pocket magnifiers and weekly pill organisers. Difficulties faced by patients with blindness in using medicines, and how they deal with these difficulties, are largely unknown and underexplored, especially in the developing world. This limits the implementation of policies and strategies to cater for the medication-related needs of visually impaired patients. The objectives of the study were to describe the challenges that patients with partial or complete blindness experienced when taking chronic medications and to identify methods used to overcome the challenges.

## Methods

### Study design

A quantitative cross-sectional descriptive study design was used, in which the researchers administered a structured questionnaire, complemented with some open-ended questions, to patients with partial or complete blindness. The questionnaire was administered by face-to-face meetings with participants who consented to data collection.

### Study site

The study was conducted at the eye clinic of Mankweng Hospital in Capricorn District, Limpopo Province, South Africa.

### Study period

The study was performed in the months of September 2017 and October 2017.

### Study population

Patients with partial and complete blindness, using chronic medications, and attending the Mankweng Hospital eye clinic between September 2017 and October 2017.

### Study sample

The study was conducted on 82 participants who met the criteria over a period of 2 months. The participants were selected using purposive sampling. In this study, a person with partial blindness, or visual impairment was defined as one who has impairment, of visual functioning, even after treatment, and/or standard refractive correction, has visual acuity (VA) of less than 6/18 to light perception, or a visual field of less than 10 degrees from the point of fixation, but who uses, or is potentially able to use, vision for the planning and/or execution of a task (Naipal & Rampersad 2018).

### Data collection

Data were collected through the administration of a structured questionnaire, complemented with some open-ended questions by the researcher to patients with partial or complete blindness. Questions were asked in the most comfortable language for each participant. Data collectors were fluent in local languages around the Mankweng area. The questionnaire was divided into four sections (A, B, C and D). Section A included demographic details. Section B covered attitudes regarding medicine use. Section C included challenges faced when taking chronic medication, and section D described methods used to overcome challenges faced. A pilot study was conducted on 10 participants to test the validity and reliability of the questionnaire, and feasibility of the study. Participants interviewed in the pilot study were excluded from the final research sample.

### Data analysis

Data were recorded on Microsoft Excel (Microsoft Corporation, Redmond, Washington, United States), and analysed by calculating percentages, using the Statistical Package for the Social Sciences (SPSS), version 22.0.0.0, software (IBM Corporation, Armonk, New York, United States).

### Ethical considerations

The study was fully approved by the Turfloop Research Ethics Committee (TREC; ref. no. TREC/126/2017:UG). The aim and objective of the study was explained to the participants prior to participation in the study. Participation in the study was voluntary, and participants were given the option to withdraw from the study at any time, without any form of penalty.

## Results

### Gender and age

A total of 82 participants were interviewed in the study, 42 male participants and 40 female participants, indicating an even gender distribution, as shown in Table 1. Sixty-two participants (76%) were over 50 years of age, as shown in Table 2. A staggering 59% of the participants were the elderly, above 60 years of age.

**TABLE 1:** Gender distribution, visual impairment, level of education and braille literacy of participants.

Demography	Male		Female		Total	
	*n*	%	*n*	%	*n*	%
Gender distribution	42	-	40	-	82	-
**Level of visual impairment**						
Some sight	34	-	30	-	64	78
No sight at all	8	-	10	-	18	22
**Highest level of education**						
No formal education	9	21.4	10	25	19	23
Primary	17	40.5	11	27.5	28	34
Secondary	10	23.8	10	25	20	24
Tertiary	6	14.3	9	22.5	15	19
**Ability to read braille**						
Yes	7	-	8	-	15	18.3
No	35	-	32	-	67	81.7

**TABLE 2:** Age range of participants.

Age range	Frequency	%
0–20 years	3	3.7
21–30 years	8	9.8
31–40 years	2	2.4
41–50 years	7	8.5
51–60 years	14	17.1
60–70 years	17	20.7
71–100 years	31	37.8

### Level of visual impairment

Among the 82 participants, 64 participants (78%) were partially blind, of whom 34 were male and 30 were female. Eighteen participants (22%) had total blindness, of whom eight were male and 10 were female, as shown in Table 1.

### Level of education

The level of education was found to be higher in female participants than in male participants. As shown in Table 1, 22.5% of female participants achieved tertiary education, whereas only 14.3% of male participants had tertiary education. A total of 23% of the participants had no formal education, and a further 34% did not go beyond primary education.

### Ability to read braille

Only 18.3% of the participants could read braille, whilst 81.7% could not, as shown in Table 1. The cost of learning how to read braille in a township is not feasible, and special schools for the blind are not yet available in Mankweng township. There are approximately six schools for the blind in the Limpopo province, and the nearest school for the blind is approximately 60 km away from Mankweng (SANBC 2015). These circumstances make it extremely difficult for blind people, who live in and around Mankweng township, to access facilities and institutions that teach braille.

### Chronic medication used

About 43% of the participants were found to be on multiple medications. These medications include diabetes medication, psychiatric medication, antiretrovirals (ARVs) and hypertensive medication, which was the most common (Table 3). About 7.32% of patients were on other medications (asthma medication or rheumatoid arthritis).

**TABLE 3:** Type of chronic medication used by participants.

Chronic medication	Frequency	%
Diabetes	12	14.63
Hypertension	16	19.51
Psychiatric	6	7.32
Antiretroviral therapy	6	7.32
Multiple	36	43.90
Other	6	7.32

### Challenges

Table 4 lists the common challenges faced by participants who self-administer their chronic medication chronic medications. Among 82 participants, 33% could not locate their medication, 26% could not identify the medication and 16% had taken incorrect medication by mistake. Participants indicated that these challenges are frequent in the early stages of therapy, but with time, they come to identify specific spots in which to keep their medication. However, challenges arise when new medications are added to their list. Errors increase when medication is taken in a rush, without paying much attention to the medication being picked up.

**TABLE 4:** Challenges when self-administering chronic medication.

Challenges faced	Yes		No	
	*n*	%	*n*	%
Could not locate medication	27	33	55	67
Could not identify medication	26	32	56	68
Wrong medicine administered by mistake	13	16	66	84
Inability to open medicine container	27	33	55	67
Did not know the correct dose	36	44	46	56
Taken wrong dose	37	33	55	67
Inability to self-administer medicine	21	26	61	74
Ever felt that incorrect amount was dispensed from the container	51	62	31	38
Spilled liquid medicine	27	33	55	67
Ever missed a dose	52	64	28	36
Ever felt difficulties with storage of medicine	49	60	33	40

Self-administration of medicines presents many challenges. Twenty-six per cent of the participants alluded to being unable to self-administer. This is usually the case with participants with total blindness but also depends on the severity of the condition in those with partial blindness. A total of 27% of the participants could not open the medication containers, 27% spilled medicines and 62% felt the incorrect amount was dispensed from the medicine container. A further 67% of the participants could open medication containers, especially solid dosage forms. Participants further explained that liquid medicines and bulk containers were more challenging. Participants have to accurately measure liquid medicines and administer the dosage without spillage.

It is worrying that 33% of the participants had taken wrong doses at some point, 44% did not know the correct dose and 64% had missed doses. This prevents benefit from the effective pharmacological therapy of the medicines. A total of 60% of the participants had encountered difficulties with the storage of medicines.

### Current methods used to overcome challenges

Table 5 depicts methods used by partially blind and completely blind patients to overcome challenges encountered when taking chronic medications. It is interesting to note that only one participant (1.2%) used pill-organising boxes, whilst a few others kept different medicines in different places or placed tactile marks on medicine packaging. A sizeable number of participants (24.48%) depended on the caregiver to overcome the challenges they faced. The caregivers would administer medication to them or supervise the administration. A majority of the participants did not have any specific methods to overcome challenges. This indicates that a majority of the participants were at a high risk of using medication in an incorrect manner.

**TABLE 5:** Current methods used to overcome self-administration challenges.

Method used	Frequency	%
Pill organiser	1	1.2
Keeping different medicines in different places	3	3.7
Placing touchable marks on medicine packaging	2	2.4
Caregiver-dependent	20	24.4
No method	56	68.3

### Preferred solutions to improve medication use

The participants indicated their preferred solutions to improve the challenges faced with medication use, as listed in Table 6. Preferred solutions included an additional explanation by the pharmacist, braille-coded patient information leaflets, and audio information. The most preferred solutions were identifying medicines by touching differentiating marks on medicine packaging and braille-coded labels.

**TABLE 6:** Preferred solutions to improve medication use and identification.

Solution	Frequency	%
Extra explanation by the pharmacist	7	8.5
Braille labelling	20	24.4
Touching differentiating mark on the drug package	29	35.4
Patient information leaflet in braille	16	19.5
Availability of information in audio format	10	12.2

## Discussion

The prevalence of partial and complete blindness in the elderly in this study was similar to a survey done by Mariotti and Pascolini (2012) in which participants of age 50 years and older represented 65% and 82% of those with visual impairment and blindness, respectively. Similarly, another study by Bourne et al. (2017) indicated a high prevalence of partial and complete blindness in the elderly and identified the main causes of visual impairment among adults over 50 years of age to be uncorrected refractive errors, cataract, glaucoma, age-related macular degeneration, diabetic retinopathy, corneal opacity and trachoma. However, a study by Keeffe et al. (2019) has revealed a significant decrease in the prevalence of blindness among older adults aged 50 years and above from about 2.7m – 3.4m (3.0%) in 1990 to about 1.7m – 2.2m (1.9%) in 2010, a decrease of 0.4m – 0.8m (0.5%) per decade. During the same period, the global age-standardised prevalence of partial blindness among older adults decreased from about 12.1m – 16.2m (14.3%) to about 9.5m – 12.3m (10.4%), a decrease of 0.4m – 2.8m (2.0%) per decade (Keeffe et al. 2019).

Similar to a study by Stevens et al. (2013), on global prevalence of vision impairment and blindness, there are likely to be more people with visual impairment than people who are blind, in a setting. The ratio of 78:22 of participants with partial blindness to participants with blindness in the current study, suggests the loss of vision has occurred progressively, possibly over the years and is possibly due to age-related conditions, such as glaucoma and cataracts.

A high percentage of participants with no or low levels of education in the study is a glimpse of the reality in Mankweng, a disadvantaged black rural township in South Africa. Patients’ level of education and literacy and ability to understand medication information may potentially affect their use of medication. A study by Davis et al. (2006) found patients with low literacy to have difficulty in understanding prescription medication labels. Davis et al. (2006) further argued that since patients are increasingly exposed to many prescription and nonprescription medications, adverse events resulting from improper medication administration are a serious concern. Furthermore, another study by Shi et al. (2019) showed medication literacy, particularly among the elderly, as a whole variable and three dimensions (of knowledge, attitude and behaviour) to be significantly associated with medication adherence. Shi et al. (2019) concluded that patients’ medication adherence could be enhanced by increasing their medication literacy level. Increasing the literacy level among patients with partial and complete blindness could be key in promoting full medication literacy and thereby enabling patients to utilise any strategies implemented that are specific to several dimensions of medication literacy.

In a developing country like South Africa, a former colony, the black majority had been politically excluded from free and compulsory education before independence, and schools for the blind, specifically for black people, were rare (Mckay & Romm 2015). However, over the years, efforts have been made to remove barriers and provide education to all previously disadvantaged communities and people, including those with disabilities. In 2008, the Kha Ri Gude Literacy Campaign was established to improve literacy in South Africa. Among other objectives, the campaign aimed to develop strategy to provide literacy in braille and basic orientation and mobility skills for adults who are blind and illiterate. Specifically, the campaign recruits, trains and deploys teachers to teach literacy and numeracy to adult learners who are blind (Mckay & Romm 2015). However, such excellent campaigns’ and initiatives’ progress need to be assessed and evaluated.

A study by Kao and Mzimela (2019) assessed Grade R in-service teachers’ knowledge of teaching and prereading skills to learners with visual impairments. The study revealed that although some of the participants showed a high level of technological knowledge, there was a tendency to teach braille as a ‘standalone’ skill and fail to integrate it with the teaching of other prereading skills to Grade R learners. Kao and Mzimela (2019) further highlighted the need for supportive in-service teacher education programmes that equip Grade R teachers of learners with visual impairments with the necessary skills to teach prereading skills. Implementing such programmes could ultimately assist patients with blindness and visual impairments to self-administer medicines by following instructions.

It is important to note that in the current study only a small percentage of the participants were totally blind compared with a large percentage that was partially blind, possibly because of age-related degenerative eye conditions. Because of this, only a few participants would have been expected to learn braille from an early age. A major concern that arises is that only a few patient information leaflets are written in braille. This is a major drawback, since those who are blind or visually impaired may not be able to use medication on their own, even if they are braille literate. A similar concern was raised by Kentab et al. (2015) in a study that explored medication use by patients with blindness in Saudi Arabia. The study found that the majority of patients with blindness (91%) were able to use the braille. However, only 18% of the patients had received drug labels in braille, and none of them had received medication information pamphlets in braille. Braille literacy should be complemented by braille-coded medication information pamphlets.

Taking multiple medications generally contributes to more challenges when it comes to self-administration in elderly patients. This could be the case with participants in the current study, where a number of them were taking multiple drugs. A study by Runganga, Peel and Hubbard (2014) investigated the extent of polypharmacy in patients receiving post-discharge transitional home care and reported similar concerns. Results of the study revealed polypharmacy (5–9 drugs) in 46.7% of the participants and hyperpolypharmacy (greater than 10 drugs) in 39.2% of the participants. Increasing numbers of medications were further associated with a greater number of comorbid conditions, a higher prevalence of diabetes mellitus, coronary heart disease and chronic obstructive pulmonary disease. It would be helpful if formulation scientists investigated the possibility of combining a number of drugs used in the treatment of conditions prevalent in the elderly into fixed-dose combinations to reduce a high pill burden and associated challenges.

It is of paramount importance to assess medication use by the visually impaired, especially elderly patients, and identify any challenges faced. Dagli and Sharma (2014) have cautioned that elderly people are at a greater risk for adverse drug reactions because of the metabolic changes and reduced drug clearance associated with ageing, and the risk is furthermore exacerbated by increasing the number of drugs used. Tamez Peña et al. (2014) advocated that medication prescription must be based on general standards of the use of medication in the elderly. Furthermore, Tamez Peña et al. (2014) stated that the main objective of medication prescription should be to decrease the use of multiple medications and increase safety and treatment adhesion.

It appears that the challenges faced by participants in this study are similar to those faced by other patients with blindness or visual impairments around the world. A study by Weeraratne et al. (2012) identified similar challenges such as inability to locate medicines (25.39%), difficulty identifying medicines and medicine containers (17.46%) and difficulty administering liquid medications (25.39%). These difficulties led to inaccurate dosing, missing doses and discontinuation of treatment prematurely.

Another study by Zhi-Han, Hui-Yin and Makmor-Bakry (2017) showed similar challenges faced by patients with visual impairment when using medication. In the study, all of the respondents perceived that self-administration of medication was a challenging task. A very high percentage of the respondents could not read prescription labels, did not know the expiry date of their own medication and did not know the name of the medication. Furthermore, comparable to results of the current study, respondents did not practise appropriate methods to store their medication and kept the unused medication. Over and above this, almost all respondents did not tell healthcare providers when they faced difficulties in handling their medication (Zhi-Han et al. 2017). Could this, in general, suggest patients’ fear of seeking help or could this be an indication of healthcare providers not encouraging patients with blindness and visual impairment to open up about challenges faced?

If medication challenges faced by patients with blindness and visual impairment are not addressed, they have the potential of greatly hindering adherence to treatment. This was proposed by Bashyal et al. (2019) in a study that investigated medication utilisation problems among the population with blindness in Nepal. The study found only 21.43% of the participants to adhere to treatment plans. A number of problems, similar to problems identified in the current study, were identified among the participants. These include spilling medication during administration, not knowing the names of medication, not receiving adequate information from dispensers and not knowing any precautions related to their medicines.

Medication challenges faced by patients with blindness and visual impairment are common worldwide, and a number of methods to overcome the challenges are recorded in literature. A study evaluating medication use patterns in patients with visual impairments in Saudi Arabia gathered a number of techniques used by participants to identify medication (Almukainzi et al. 2020). Such techniques included identifying the size and shape of the formulation by touching the external packaging, identifying the surface roughness of the medication and/or the containers and smelling product odours. Other participants used techniques such as adding a raised sticker or tearing parts of carton boxes, printing braille stickers on each medicine container and using audible records for each medication prescription (Almukainzi et al. 2020). However, participants in the current study did not use these techniques.

In another study by Naik, Cacodcar and Dhupdale (2018) exploring the challenges faced by patients with visual disabilities in the use of medication, the participants resorted to using various strategies to overcome challenges. The most commonly used coping measures amongst participants were keeping medications in specific places and identifying separate drugs by feeling the shape of the container.

A number of guidelines for eye health workers and other helpers to assist patients with blindness and visual impairment are available. However, these guidelines are general and not specific to medication use. Some of the general guidelines and measures listed by Stevens (2003) include writing any information provided to patients with blindness and visual impairment in readable size and font. As well, information should be passed on to an attending sighted carer for future reference. Further, anyone willing to offer help is encouraged to ask first before offering help and should not be offended if the help is refused.

The participants in the current study seemed unaware of simple methods to administer medicines. Could this indicate a lack of intervention by healthcare professionals? However, the participants were very eager to learn new methods to improve medication use and overcome challenges. Perhaps counselling and training patients with blindness and visual impairment on known simple strategies could reduce challenges faced when using medicines. Medicine manufacturing companies could consider printing patient information leaflets additionally in braille.

## Conclusion

The study assessed challenges faced by people with visual impairments in Mankweng township in South Africa. A majority of participants were the elderly who had lost sight partially, possibly because of age-related conditions, and were on multiple chronic medications. Challenges faced by participants included not being able to locate and identify medication, missing doses, inaccurate dosing and spilling medicines. Furthermore, only a few participants could read braille. However, most medicine labels and patient information leaflets currently utilised by the participants, are not written in braille. Some participants relied on caregivers to administer medication; a few others use methods such as pill organisers. Other participants kept medicines in different places and would place tactile markings on medicine packages. It was noted that close to 70% of the participants did not have specific methods to overcome challenges, thereby exposing them to wrong use of medicines. Literature reveals that the challenges faced by the blind and partially blind in the current study are similar to challenges faced globally by the blind and visually impaired. Various known, simple methods to overcome challenges could be shared with the blind and visually impaired in Mankweng to lessen the challenges. Awareness needs to be raised to healthcare policymakers of underexplored challenges faced by patients with blindness and visual impairments, in use of medicines. The Department of Health could engage with relevant associations for blind and visually impaired persons, to discuss solutions and train healthcare professionals, such as nurses, on how to communicate uniquely with blind and visually impaired patients. Programmes may be set up at clinics, hospitals and healthcare centres to teach people with visual impairments simple and inexpensive methods to administer medications.
